# Association between the development of bronchopulmonary dysplasia and platelet transfusion: a protocol for a systematic review and meta-analysis

**DOI:** 10.3389/fped.2023.1049014

**Published:** 2023-06-09

**Authors:** Roberto Chioma, Stefano Ghirardello, Krzysztof Włodarczyk, Joanna Ulan-Drozdowska, Antonio Spagarino, Marta Szumska, Klaudia Krasuska, Joanna Seliga-Siwecka, Roy K. Philip, Niazy Al Assaf, Maria Pierro

**Affiliations:** ^1^ Department of Woman and Child Health and Public Health, Neonatology Unit, Università Cattolica del Sacro Cuore, Rome, Italy; ^2^ Department of Woman and Child Health and Public Health, Neonatology Unit, Ospedale San Matteo, Pavia, Italy; ^3^ Main Library, Medical University of Warsaw, Warsaw, Poland; ^4^ Neonatal and Intensive Care Department, Medical University of Warsaw, Warsaw, Poland; ^5^ Neonatal Intensive care Unit, University of Ferrara, Ferrara, Italy; ^6^ Division of Neonatology, Department of Pediatrics, University Maternity Hospital Limerick and Medical School University of Limerick, Limerick, Ireland; ^7^ M. Bufalini Hospital, AUSL Romagna, Cesena, Italy

**Keywords:** preterm infant, NICU, platelet transfusion, bronchopulmonary dysplasia, chronic lung disease of prematurity

## Abstract

**Background:**

There is a lack of consensus on the management of thrombocytopenia in preterm infants, and the threshold for prophylactic platelet transfusion varies widely among clinicians and institutions. Reports in animal models suggested that platelets may play a relevant role in lung alveolarization and regeneration. Bronchopulmonary dysplasia (BPD) is a severe respiratory condition with a multifactorial origin that affects infants born at the early stages of lung development. Recent randomized controlled trials on the platelets count threshold for prophylactic transfusions in preterm infants with thrombocytopenia suggest that a higher exposition to platelet transfusion may increase the risk of BPD. Here, we report a protocol for a systematic review, which aims to assist evidence-based clinical practice and clarify if the administration of platelet products may be associated with the incidence of BPD and/or death in preterm infants.

**Methods:**

MEDLINE, Embase, Cochrane databases, and sources of gray literature for conference abstracts and trial registrations will be searched with no time or language restrictions. Case–control studies, cohort studies, and nonrandomized or randomized trials that evaluated the risk for BPD and/or death in preterm infants exposed to platelet transfusion will be included. Data from studies that are sufficiently similar will be pooled as appropriate. Data extraction forms will be developed *a priori*. Observational studies and nonrandomized and randomized clinical trials will be analyzed separately. Odds ratio with 95% confidence interval (CI) for dichotomous outcomes and the mean difference (95% CI) for continuous outcomes will be combined. The expected heterogeneity will be accounted for using a random-effects model. Subgroup analysis will be performed based on *a priori*-determined covariate of interest. In case of sufficient homogeneity of interventions and outcomes evaluated, results from subgroups of studies will be pooled together in a meta-analysis.

**Discussion:**

This systematic review will investigate the association of BPD/death with platelet components administration in preterm infants, and, consequently, it will provide reliable indications for the evidence-based management of premature patients with thrombocytopenia.

## 1. Introduction

Bronchopulmonary dysplasia (BPD), also known as chronic lung disease (CLD) of prematurity, is a severe respiratory condition with a multifactorial origin that affects infants born at the early stages of lung development. BPD is the commonest respiratory morbidity among very premature infants, leading to short- and long-term pulmonary and non-pulmonary complications. The arrest in lung development, due to prematurity itself, is considered a prerequisite to the development of lung damage (1, 2). In addition, the role of several pro-inflammatory prenatal and postnatal pathogenic noxae is well-known, all driving to lung injury through chronic inflammation (3).

Recently, a new insight into the possible interaction between platelet transfusion and impairment of lung parenchyma development was provided by the pivotal PlaNeT-2 trial (4). In this study, a more liberal platelet transfusion threshold (50 × 10^9^/L) for prophylactic use increased the risk of BPD, compared to a more restrictive threshold (25 × 10^9^/L). The authors hypothesized a possible role of pro-inflammatory injury mediated by platelet product-derived bioreactive components (5), augmenting oxidative stress and aberrant angiogenesis. However, the PlaNeT-2 trial was not primarily designed to evaluate the relationship between platelet transfusions and BPD, as the main outcome was a composite of death or major bleeding. Moreover, the results for secondary outcomes could not be adjusted for multiplicity. Given the lack of strong evidence, it is a matter of priority to synthesize the available data from the literature through a systematic review and meta-analysis.

While thrombocytopenia is frequently encountered among preterm infants, there is a lack of consensus on its management, and the threshold for prophylactic platelet transfusion varies widely among clinicians and institutions (6–8). Several studies suggest that platelet products may have systemic pro-inflammatory consequences and damage various organs, including the lung (4, 5). There is a growing body of research about the interaction between platelet biogenesis and pulmonary development (9, 10). Animal studies suggest that up to 50% of circulating platelet biogenesis could be in the lungs in mammals (11). Recent reports in animal models also suggested that platelets may play a relevant role in lung alveolarization and regeneration (9, 10).

Here, we report a protocol for a systematic review, which aims to assist evidence-based clinical practice and clarify if the administration of platelet products could be associated with the development of BPD and/or death in preterm infants.

## 2. Methods and analysis

### 2.1. Protocol and registration

The reporting guidelines of the Preferred Reporting Items for Systematic Reviews and Meta-Analysis for Protocols 2015 (PRISMA-P) were followed for the development of the present protocol. The filled PRISMA-P checklist is available as Supplementary material. This protocol is registered in the PROSPERO International Prospective Register of Systematic reviews (no. CRD42021279329). The resulting review will follow the updated PRISMA statement and will report important amendments to the original protocol.

### 2.2. Population, intervention, comparison, and outcomes questions

This project aims to answer two population, intervention, comparison, and outcomes (PICO) questions:

1. Are preterm infants receiving platelet transfusions at higher risk of BPD compared to those who did not?
2. Does a liberal threshold for prophylactic platelet transfusion increase the risk of developing BPD in preterm infants?

### 2.3. Study selection

Studies will be selected by the following criteria.

#### 2.3.1. Population

##### 2.3.1.1. Inclusion criteria

(1) Preterm babies born <32 weeks of gestational age (GA).

##### 2.3.1.2. Exclusion criteria

(1) Infants with lung malformations, or related lung morbidity not related to prematurity.
(2) Infants with inborn platelets disorders.
(3) In case of relevant studies including populations with mixed GA, we will contact the study authors to obtain data from all patients with a GA below 34 weeks. If the authors cannot provide this information, the study will be excluded.

#### 2.3.2. Type of studies

##### 2.3.2.1. Inclusion criteria

This systematic review will include case–control studies, cohort studies, and nonrandomized or randomized trials that evaluated platelet transfusion in preterm infants. Both prospective and retrospective studies will be included. This data analysis will not combine randomized controlled trials (RCTs) with observational and nonrandomized studies.

##### 2.3.2.2. Exclusion criteria

This systematic review will not include case series, qualitative thematic analysis, narrative reviews, editorials, systematic reviews, or expert opinions.

#### 2.3.3. Type of intervention

##### 2.3.3.1. Inclusion criteria

We will include preventive and rescue platelet transfusion regardless of the initial platelet count. Preventive platelet treatment refers to transfusion in the case of increased risk of bleeding, while rescue treatment refers to transfusion in the case of active bleeding. We will not limit preventive transfusion by platelet threshold, although the threshold and the pre-transfusion platelet count will be recorded and serve as items for subgroup analysis. We will not limit the inclusion of the study based on reason for platelet transfusion. Therefore, any indication will be admissible. Whether or not the indication for platelet transfusion is described in the study, it will be recorded and assessed in subgroup analysis or meta-regressions, if a sufficient number of studies are found.

##### 2.3.3.2. Exclusion criteria

Studies evaluating other interventions in case of risk of bleeding or active bleeding will be excluded.

#### 2.3.4. Type of comparator

We will include studies having control groups assigned to placebo or no intervention. The comparator may also be represented by different thresholds of platelet count in the case of preventive transfusion.

#### 2.3.5. Timeframe

The primary outcome (BPD incidence) will be assessed at 36 weeks of post-menstrual age (PMA). Transfusion-related adverse events will be considered if they occurred up to 6 h from the platelet product administration. The secondary medium-term outcomes will be regarded as starting from birth to discharge from the neonatal intensive care unit (NICU). Long-term outcomes will be considered up to 6 years of age.

If the timeframe of the listed outcomes is not specified, we will contact the authors of the study to obtain the specific data. If the authors will not be able to provide this information, the study will be excluded.

#### 2.3.6. Setting

The study setting will be NICU stay.

#### 2.3.7. Language and publication time

We will not apply any time or language restrictions.

### 2.4. Outcome measurements

#### 2.4.1. Primary outcome

Primary outcome extracted from articles will focus on the incidence of BPD, defined as oxygen dependency at 36 weeks PMA and further classified by disease severity categories according to the most adopted definitions (12, 13). However, we will not select the studies based on BPD definition. We will include any BPD definition and then run subgroup analysis based on that.

#### 2.4.2. Secondary outcomes

The secondary outcomes are grouped in immediate transfusion-related complications and medium-term outcomes, as follows.

*Immediate transfusion-related complications*

(1) Incidence of any adverse event possibly related to platelet transfusion. Adverse events will be classified as serious or nonserious; expected or unexpected; and study-related, possibly study-related, or not study-related.
(2) Pulmonary hypertension (14).
(3) Incidence of lung-related transfusion complications, transfusion-associated lung injury (15), defined as acute respiratory distress during or within 6 h of blood component, in the absence of temporally associated risk factors for respiratory distress syndrome.
(4) Mortality within 24 h from platelet transfusion.

*Medium-term outcomes:*

(1) Duration of ventilation: invasive, noninvasive, and total duration of ventilator dependency.
(2) Duration of low-flow oxygen dependency: days spent on low-flow supplemental oxygen (below 3 L/min); can be administered through nasal cannula or oxygen hood.
(3) Postnatal steroids treatment, including, but not limited to, betamethasone, hydrocortisone, dexamethasone, methylprednisolone, and prednisolone.
(4) Duration of hospital stay.
(5) Discharge with home oxygen or home ventilation with or without tracheostomy.
(6) Incidence of retinopathy of prematurity, classified in five stages, ranging from mild (stage I) to severe (stage V). Aggressive-posterior ROP will be also recorded (16).
(7) Incidence and type of treatment for retinopathy of prematurity.
(8) Incidence of intraventricular hemorrhage, defined and classified according to either to Papile or Volpe grading (17, 18).
(9) Incidence of periventricular leukomalacia, defined as either focal (“cystic PVL”) or diffuse (“non-cystic PVL”) injury (19).
(10) Incidence and treatment of necrotizing enterocolitis defined according to Bell staging or modified Bell staging (20, 21).
(11) Incidence and treatment of patent ductus arteriosus, defined as hemodynamically significant according to echocardiographic criteria chosen by the authors (22).
(12) Incidence of early-onset and late-onset sepsis, defined as clinical deterioration with positive cultures (23).
(13) Mortality during the NICU stay.
(14) Mortality during the NICU stay due to respiratory morbidity, in case it is separately described by the authors.

*Long-term outcomes (up to 6 years):*

(1) Somatic growth.
(2) Admissions to the hospital.
(3) Number and type of infections.
(4) Neurological development defined by Bayley-Ill scale at 2 years of corrected age (24).
(5) Need for transfusion.
(6) Survival.

We aim to obtain a comprehensive evaluation of possible complications or medium-term outcomes deriving from platelet transfusion. However, as recommended, studies not respecting the present definitions or reporting different outcomes will not be excluded. Eventually, we will specify in the review secondary outcomes added to the original list.

If the information is provided, subgroup analysis will evaluate the possible association of volume and rate of platelet transfusion with the different outcomes.

### 2.5. Search strategy

The MEDLINE, Embase, and Cochrane databases have been searched for this systematic review, following a standardized strategy developed using a standardized set of keywords and operators, with the consult of a research librarian (KW). We have not applied any other filtering or restrictions to the search strategy.

Additional strategies included manually reviewing reference lists from key articles that met our eligibility criteria and use of PubMed's “related articles” feature. Studies included in relevant systematic reviews may also be used if they satisfy our eligibility criteria.

The search query is as follows:

(((platelet[tiab] OR platelets[tiab] OR plasma[tiab] OR plasmas[tiab] OR FFP[tiab]) AND (transfusion[tiab] OR transfusions[tiab] OR infusion[tiab] OR infusions[tiab])) OR “Platelet Transfusion”[Mesh] OR “Plasma”[Mesh]) AND (infant[tiab] OR infants[tiab] OR newborn[tiab] OR newborns[tiab] OR neonate[tiab] OR neonates[tiab] OR neonatal[tiab] OR postnatal[tiab] OR “Infant”[Mesh] OR “Intensive Care Units, Neonatal”[Mesh]) NOT (review[pt] OR “systematic review”[pt] OR “meta-analysis”[pt] OR “case reports”[pt] OR editorial[pt] OR letter[pt] OR comment[pt]) NOT (“animals”[mesh] NOT “humans”[mesh])

### 2.6. Data management

The results of the search will be uploaded to an Internet-based software program facilitating the study selection process (DistillerSR®, Ottawa, Canada). Based on the inclusion and exclusion criteria, screening questions and forms will be developed and tested for levels 1 (title and abstract screening) and 2 (full-text screening). The full-text articles for level 2 screening will be uploaded with screening questions to DistillerSR. We will conduct a calibration test before each screening step to pilot and refine the screening questions.

### 2.7. Study selection process

For feasibility reasons, the references will be divided into two sequential groups. Two authors will independently assess each group for titles and abstracts, for a total of four reviewers.

In level 1, articles’ titles and abstracts will be screened by the two independent authors using an initial screening questionnaire. In level 2, all the references retained will undergo full-text screening to select those matching our eligibility criteria. To ensure adequate inter-reviewer agreement, calibration exercises will be conducted on 20 random articles for each screening level. An agreement between authors will be needed to include a reference to the following level. When consensus cannot be reached, a third author (MP) will intervene to resolve the conflict. Where necessary, study authors will be contacted to obtain additional information to resolve eligibility issues. In the case of exclusion of trials in level 2, the reasons will be recorded. The review authors will be unblinded to the study authors or institutions or the journal titles.

### 2.8. Data extraction

Forms for the data extraction will be designed *a priori* and pilot-tested using a standardized extraction form on DistillerSR® by our team. The data extraction will be performed by two independent reviewers using DistillerSR®'s quality control function. To ensure that the approach to data charting will be consistent with the review questions and aim, the extraction forms will be piloted on five random articles. Each reviewer will chart half of these articles and audit the other half, consulting a third independent reviewer in case of conflicts. Results will be discussed by the team, and the data extraction forms will be updated throughout the process, to include other aspects of the intervention not considered *a priori*.

Table 1 will report the following extracted data:

(1) Lead author, year of publication, and country of origin.
(2) Sample size (total and per group).
(3) Design of the study.
(4) Inclusion and exclusion criteria.
(5) Setting.
(6) Definition of BPD.

Tables 2 (observational studies), 3 (interventional, nonrandomized studies), and 4 (interventional, randomized studies) will report the following extracted data:

(1) Purpose of study/study aims.
(2) Characteristics of the population (including the starting respiratory support).
(3) Details of platelet transfusion (rescue or preventive, threshold for prophylactic transfusion, number of interventions, and exact postnatal day of platelet transfusion).
(4) Results reported (including raw numbers, summary statistics, and adjusted analysis where available).
(5) Outcomes of interest, as defined above.

The corresponding author will be contacted a maximum of three times for these articles in which data cannot be extracted. The study will be excluded if the author will not be able to provide this information.

### 2.9. Risk of bias assessment

Two authors will independently assess the methodological quality in cohort and case–control studies, using the ROBINS-I (Risk Of Bias In Non-randomized Studies—of Interventions) Scale. According to ROBINS-I, after the evaluation of six different bias domains (i.e., bias due to confounding, bias in the selection of participants, etc.), studies will be classified as low, moderate, serious, critical, and unknown risk of bias. In case of conflicts on individual and total scores of the ROBINS-I scale, a third author will intervene for resolution. The risk of bias of randomized controlled trials will be assessed by the Cochrane risk-of-bias tool. The risk of bias will be evaluated as low, high, or unclear in each domain (allocation sequence, allocation concealment, blinding of participants and outcome assessors, incomplete outcome data, selective outcome reporting, and other potential sources of bias). Any possible discrepancy during the data extraction process and evaluation of risk bias will be discussed and solved among all reviewers.

### 2.10. Data analysis

Means and SDs or frequency and percentages will be used to present summary data for each study, as appropriate. The mean difference [95% confidence interval (CI)] or standardized mean differences (95% CI), if different scales of measurement are used, will be calculated for quantitative outcomes. The method of Wan et al. will be utilized to estimate the mean and SD in studies reporting median and range or interquartile range for quantitative variables (25). The odds ratio (OR) with 95% CI will be calculated for nominal outcomes from the data obtained from the studies. Data about ORs adjusted for potential confounders will be extracted from studies performing this analysis. For this meta-analysis, OR was chosen for all types of articles, including RTCs. Using the same effect size measure for all the types of studies may help evaluate the effect of the study design on the association. For example, we may see the influence of the study design on the result comparing the OR of randomized controlled trials’ meta-analysis and the OR of cohort meta-analysis.

Meta-analysis will be performed if we find at least two suitable studies, using comprehensive meta-analysis software (Biostat, Inc., Englewood, CO, United States). A narrative description of the study results will be provided if the number of studies is sufficient to carry out a meta-analysis, pooling data from sufficiently similar studies.

Meta-analysis will be performed separately for observational studies and nonrandomized trials and randomized controlled trials. Moreover, the rescue and the preventive administration of platelet transfusions will be analyzed separately, since the underlying clinical settings are too dissimilar to be combined.

A random-effects model will permit us to account for the expected heterogeneity, between studies as well as within studies. We chose our model *a priori*, as a formal test for homogeneity based on the *Q* and *I*^2^ statistics may not always be fully appropriate for choosing the analysis method (26, 27). We will not adopt the fixed-effects model, since we cannot assume that there will only be sampling error (28, 29). Furthermore, the random-effects model is more suitable for generalizing the analysis to other populations.

To evaluate statistical heterogeneity, we will use Cochran's *Q* statistic and the *I*^2^ statistic, derived from *Q* and explains the proportion of variation that is due to heterogeneity beyond chance. *I*^2^ greater than 50% would indicate significant heterogeneity, requiring the following analysis. Univariate random-effects meta-regression (method of moments) will be executed, in the case that more than 10 studies will be included, to explore differences among studies that might be expected to influence the effect size. We will consider statistically significant a probability value inferior to 0.05 (0.10 for heterogeneity). Subgroup analysis will be performed as well, based on the *a priori-*determined covariate of interest. In order to better interpret heterogeneity, we will also report the prediction intervals, so that treatment effects in future settings can be predicted.

The predicted sources of variability defined to drive the subgroup analysis and/or meta-regression will be: (i) disease of pregnancy (chorioamnionitis, placental dysfunction, diabetes, etc.); (ii) gestational age; (iii) birth weight; (iv) sex; (v) platelet initial count; (vi) reason, number, and timing of transfusion; (vii) ongoing treatments to prevent BPD (i.e., postnatal steroids, diuretics, bronchodilators, pulmonary vasodilators, vitamin A); (viii) site of bleeding (lung, intraventricular of gastrointestinal); (ix) neonatal morbidity (complication of prematurity, respiratory infections, late-onset sepsis, pulmonary hypertension, poor growth, difficulty feeding, developmental delay); (x) the oxygen saturation target defined as low target (85%–89%) or high target (91%–95%); (xi) threshold for platelet transfusion; and (xii) definition of established BPD and severity of BPD (moderate vs. severe). The mixed-effects model will guide the subgroup analysis: a random-effects model will be used to combine studies within each subgroup, and a fixed-effects model will be used to combine subgroups and yield the overall effect. We do not assume that the study-to-study variance is the same for all subgroups, and its value is not pooled across subgroups but computed within subgroups. Egger's regression test and funnel plots will be utilized to assess publication bias. We defined the most likely subgroup analysis. In case some other variables may show a significant difference, they will be used for subgroup analysis.

In order to assess the impact of a specific study on the overall conclusion, we will also perform a cumulative meta-analysis.

### 2.11. Data synthesis

The characteristics and findings of the included studies will be summarized and described with a systematic narrative synthesis, with the information presented in the text and tables. Moreover, a narrative synthesis of the studies that cannot be integrated in the meta-analysis will be offered. The Grading of Recommendations Assessment, Development, and Evaluation (GRADE) working group methodology will be used to estimate the quality of evidence for all outcomes (30–32). The domains of risk of bias, consistency, directness, precision, and publication bias will be used to judge the quality of evidence. It will be assessed as high (further research is very unlikely to change our confidence in the estimate of effect), moderate (further research is likely to have an important impact on our confidence in the estimate of effect and may change the estimate), low (further research is very likely to have an important impact on our confidence in the estimate of effect and is likely to change the estimate), or very low (very uncertain about the estimate of effect).

### 2.12. Missing data

We will not exclude studies because of missing summary data. We will include the study in the review, and then discuss the potential implications of its absence from the meta-analysis. If the details of the study-level characteristics needed for subgroup analysis or meta-regressions are not available, we will contact the study authors for more information. In case the authors are not willing or not able to provide the information required, we will discuss that as a limitation.

## 3. Discussion

The impact of platelet transfusion on the short- and long-term outcome, including BPD, is yet to be determined. Similarly the best threshold for platelets transfusion in non bleeding infants has not yet be defined. In 2020, a protocol for a systematic review on platelet transfusion for neonates was published (33). However, the primary objective differs in assessing evidence concerning the best threshold for platelet transfusion to reduce mortality, bleeding, and major morbidity among neonates with thrombocytopenia. The study has not been published yet. Our study differs from the one proposed by Liu et al. in the scope and the methods. Our aim is to evaluate a possible impact of platelet transfusion on the incidence of BPD in preterm infants. Hematological triggering as a contributor in the development of BPD has already been demonstrated through red cell concentrate (RCC) transfusions increasing the incidence of BPD in preterm infants, including its cumulative impact (34). While platelet transfusions are less frequent compared to RCC among preterm infants, demonstrating an association of platelet transfusion in the etiopathogenesis of BPD would be of considerable clinical value.
